# Development of an imidazole salt catalytic system for the preparation of bis(indolyl)methanes and bis(naphthyl)methane

**DOI:** 10.1371/journal.pone.0216008

**Published:** 2019-04-25

**Authors:** Xu Wang, Courtney C. Aldrich

**Affiliations:** 1 Department of Synthetic Medicinal Chemistry, Institute of Materia Medica, Chinese Academy of Medical Sciences and Peking Union Medical College, Beijing, China; 2 Department of Medicinal Chemistry, University of Minnesota, Minneapolis, Minnesota, United States of America; Queen's University Belfast, UNITED KINGDOM

## Abstract

Imidazolium salts are shown to catalyze the rapid room temperature reaction of indoles or naphthol with aldehydes to provide bis(indolyl)methanes or bis(naphthol)methane in excellent yields and the reaction proceeds optimally in dichloromethane with no base additives. The reaction exhibits a broad substrate tolerance and occurs through nucleophilic activation of the indoles and naphthols through a cation–π interaction.

## Introduction

**Fig 1:**
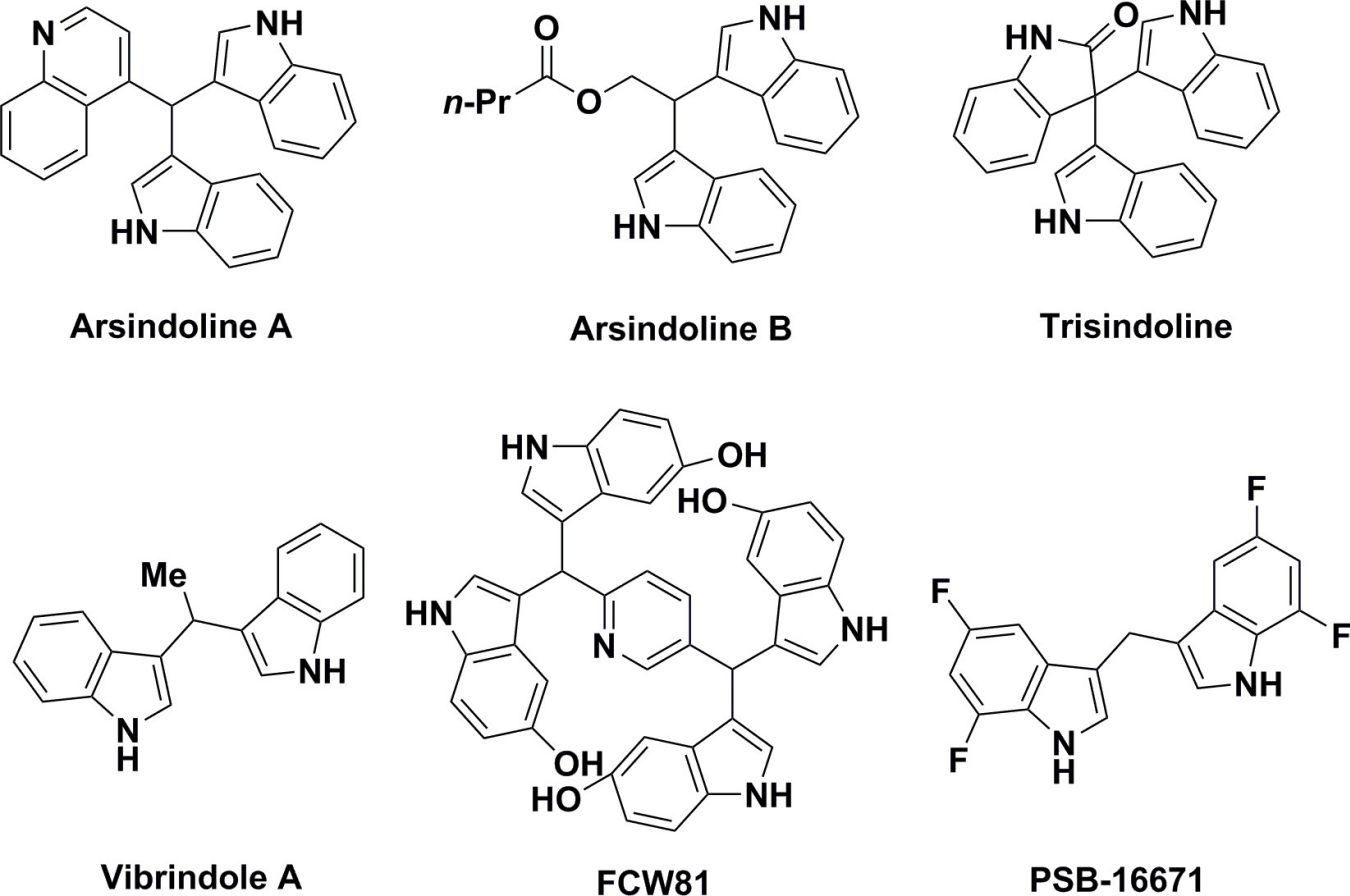
Structures of biologically active bis(indolyl)methane alkaloids.

Bis(indolyl)methane and its derivatives constitute a structurally fascinating and important class of heterocyclic compounds present in many natural products isolated from marine and terrestrial organisms (Fig 1).[1, 2] These compounds are a rich source of antitumor and antibacterial agents.[3–5] For instance, Gu and co-workers isolated two new indole alkaloids, arsindoline A and B with promising antitumor activities from a marine-derived Aeromonas bacterial strain CB101.[6] In 1994, Kobayashi and co-workers isolated trisindoline, an antibiotic indole trimer from a *Vibrio* sp. living symbiotically within the marine sponge *Hyrtios altum*.[7] Though vibrindole A was isolated from a natural source in 1994, it has been known as a synthetic product since 1963.[8, 9] Recently, Li and co-workers found the tetraindole compound, FCW81, which displayed efficacy in a xenograft model of human breast cancer by inhibiting growth and more importantly blocking cancer cell metastasis.[10, 11] In 2017, Müller and co-workers reported the bis(indolyl)methane alkaloid, PTS-16671 as a potent GPR84 agonist (EC_50_ 41 nM) that demonstrated increased stability relative to their initial lead compound.[12]

Motivated by the above-mentioned pharmacological activities of bis(indolyl)methanes, many synthetic methods have been described in the literature for preparing this class of compounds. The traditional methods of synthesizing bis(indolyl)methanes involve the activation of aldehydes using Brønsted acids [13, 14] or Lewis acids [15–21]. However, the catalysts employed are moisture sensitive and are easily decomposed or deactivated in the presence of even a small amount of water. In recent years, many new catalysts have been used to synthesize bis(indolyl)methanes including ammonium salt or borate salts [22, 23], ionic liquids [24, 25], iodine [26], heterogeneous nanoparticles [27–29] and enzymes [30]. However, these methods involve use of harsh reaction conditions as well as toxic or expensive reagents. Moreover, the substrate scope was not thoroughly explored for many of the reported reaction conditions. Development of a waste-free synthetic protocol would be of great use for the economical and practical laboratory synthesis of these compounds. In particular, any new method should ideally demonstrate a broad substrate scope in order to explore the medicinal chemistry of this scaffold.

On the other hand, to the best of our knowledge, the reaction mechanism of bis(indolyl)methanes synthesis described is based on the activation of the electrophile aldehyde rather than the nucleophile indole. If the synthesis of such compounds can be achieved by activation of the nucleophile, then we hypothesize the substrate scope can be expanded to include other electron-rich aromatics such as naphthol.

**Fig 2:**
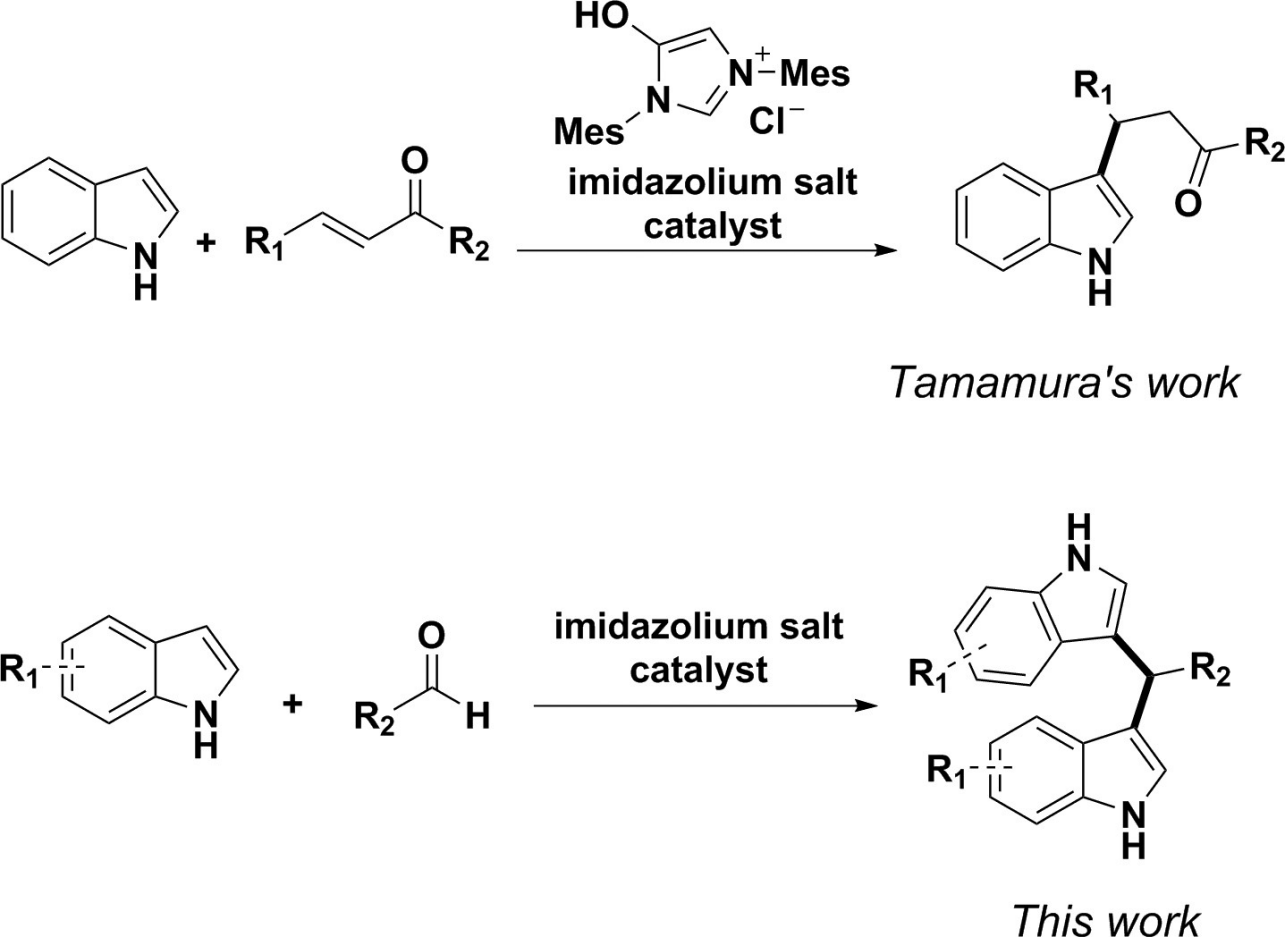
Our strategy for bis(indolyl)methane synthesis.

Imidazolium salts (and analogues) have been studied as organocatalysts for the double addition of alcohol to an aldehyde.[31, 32] In 2014, Tamamura and co-workers reported a simple method to access 3-substituted indoles employing an imidazolium salt that catalyzed Friedel-Crafts type conjugate additions.[33] The reactions were carried out under mild condition, without bases, solvents or formation of *N*-heterocyclic carbenes (NHCs). Through detailed mechanistic studies, the potential mechanism was explained through the dual activation of indole by the cation-π interaction of imidazolium cation with indoles and Lewis base activation by the chloride anion derived from the imidazolium salts. Although acidic imidazolium species have been used as catalysts to afford bis(indolyl)methanes, the mechanism is likely Brønsted acid mediated.[24, 34–36] To the best of our knowledge, the imidazolium salt-catalyzed direct addition process to simple ketones or aldehydes has not yet been reported by cation-π interactions. We thus set out to extend this reaction to efficiently construct bioactive bis(indolyl)methanes employing aldehydes as electrophiles (Fig 2).

## Results and discussion

**Fig 3:**
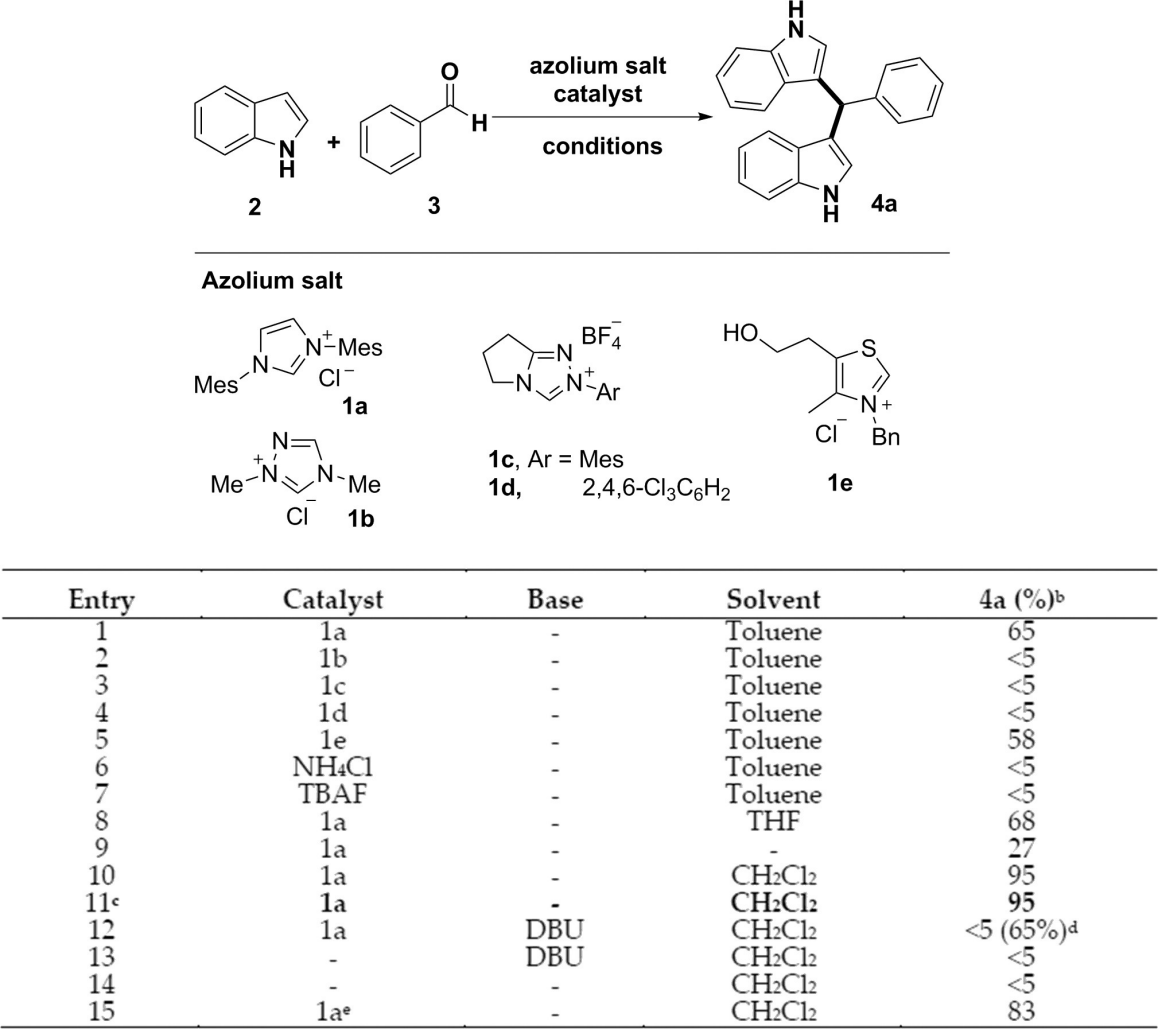
Optimization of the reaction conditions^a^.

^a^ Reaction conditions: A mixture of aldehyde (0.11 mmol, 1.1 equiv), catalyst (10 mol%, 0.2 equiv), indole (0.20 mmol, 2.0 equiv), base (0.11 mmol, 1.1 equiv) in solvent (1.0 mL) was stirred at room temperature for 1h. ^b^ Isolated yield after flash chromatography (the yields were calculated based on the equivalent of indole). ^c^ The amount of aldehyde used is 0.10 mmol (1.0 equiv). ^d^ The yield in parentheses is the yield of the benzoin reaction. ^e^ The amount of catalyst **1a** is 5 mol%.

We first undertook the screening of the azolium catalysts, including imidazolium, triazolium and thiazolium salts. Results from our catalyst evaluation are shown in Fig 3. In the absence of base, catalyst **1a** and **1e** afforded the desired product **4a** in moderate yields (entries 1, 5), while only trace amounts of product were obtained with triazolium catalysts **1b**, **1c** and **1d** (entries 2, 3, 4). The result shows that the nature of the azolium salts is critical: imidazolium and thiazolium salts are effective catalysts, whereas the triazolium salt proved to be unproductive. Based on these findings, we further investigated other reaction parameters, such as ammonium salts, solvent and base, in order to achieve a higher chemical yield. Examination of a range of ammonium salts revealed that ammonium chloride and tetrabutylammonium fluoride did not promote the reaction (entries 6, 7). The use of other solvents, such as tetrahydrofuran and dichloromethane, resulted in enhanced yields with dichloromethane affording the desired compound **4a** in an impressive 95% isolated yield (entries 8, 10). The equivalent ratio of compounds **2** and **3** was adjusted to 2:1 that is a more proper condition for maximizing the usage of reagents and the yield remained unchanged (entry 11). The yield was greatly reduced under neat conditions (entry 9). The reaction was also found to be incompatible with an amine base such as 1,8-diazabicyclo [5.4.0]undec-7-ene (DBU), which led to generation of the *N*-heterocyclic carbene of **1a** and provided 2-hydroxy-1,2-diphenylethanone as a major product through the process of benzoin reaction as reported previously and only trace amounts of the desired product (entry 12).[37] This result indicates that the weak alkalinity of indole does not induce NHC formation, unlike other organic bases. Thus the product is formed by a direct addition reaction (conjugate acid of DBU: pKa 12.0, conjugate acid of indole: pKa 0.4). As a negative control, we confirmed the reaction did not occur in the absence of the catalyst **1a** (entry 14). Decreasing the catalyst loading from 10 mol% to 5 mol% reduced the yield from 95% to 83% (entry 15) demonstrating 10 mol% is required to achieve optimal conversion.

**Fig 4:**
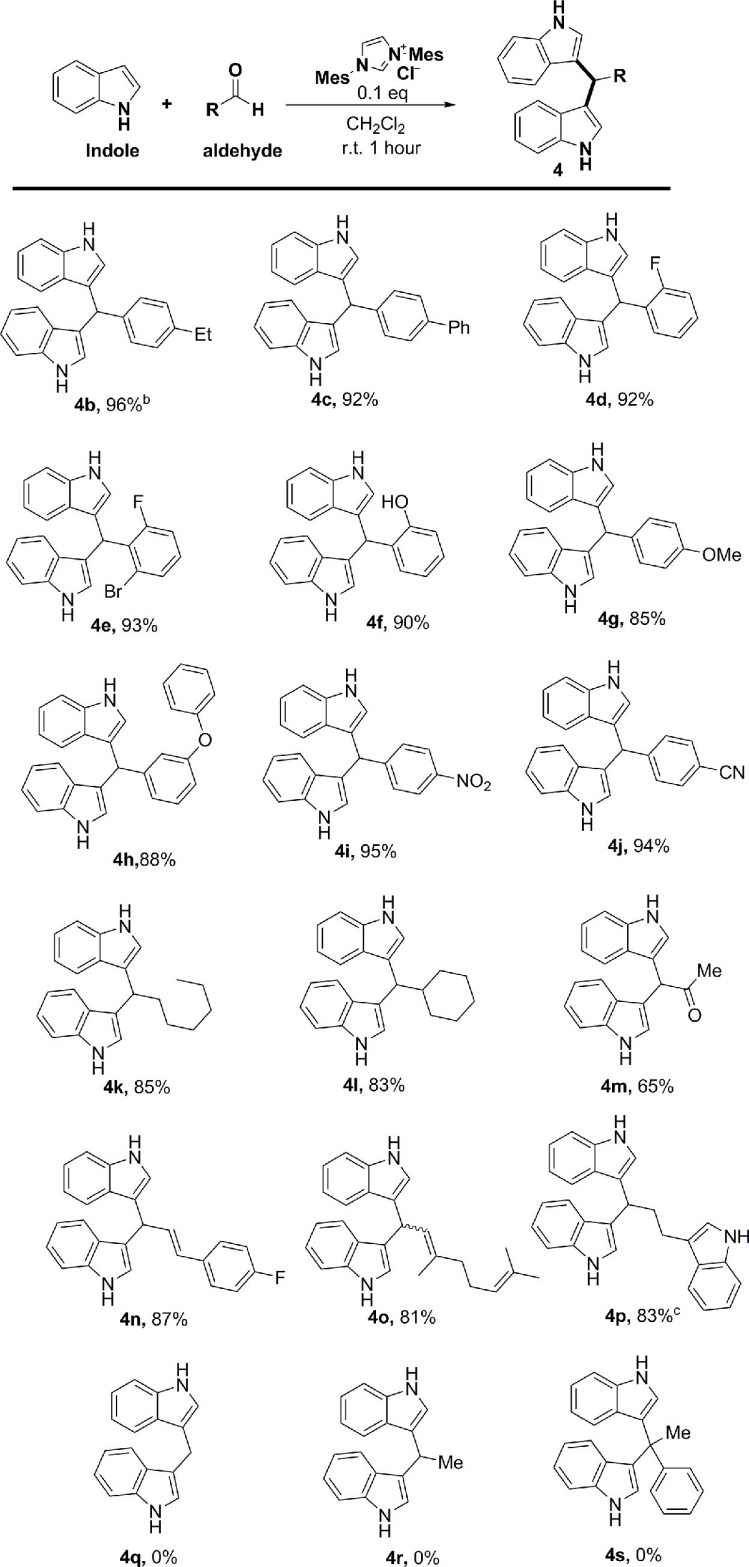
Scope of aldehydes^a^.

^a^ Reaction conditions: A mixture of aldehyde (0.5 mmol, 1.0 equiv), catalyst (10 mol%, 0.2 equiv), indole (1.0 mmol, 2.0 equiv), in solvent (5.0 mL) was stirred at room temperature for 1h. ^b^ Isolated yield after flash chromatography. ^c^ The amount of indole used is 1.5 mmol.

With the optimal conditions in hand, we next sought to explore the scope of the aldehydes in this new imidazolium salt-catalyzed Friedel-Crafts type reaction. As shown in Fig 4, a diverse array of electron-donating and electron-withdrawing benzaldehydes with a variety of functional groups (ethyl, phenyl, halide, hydroxyl, methoxy, phenoxy, nitro and cyano) performed well in this dual-addition reaction. The corresponding products (**4b**–**4g**) were isolated in excellent yields ranging from 85–96%. Notably, this method was compatible with aliphatic aldehydes (heptanal, cyclohexylcarbaldehyde, pyruvaldehyde), giving the desired products in moderate yields (**4k**–**4m**). Moreover, even unsaturated aldehyde substrates (cinnam aldehyde, citral) reacted through selective addition to carbonyl groups over conjugate addition (**4n** and **4o**). However, acrolein can give a triindole substituted product through conjugate addition and direct addition. While our method is effective for alkyl and aromatic/heterocyclic aldehydes, it fails for formaldehyde, acetaldehyde and acetophenone.

**Fig 5:**
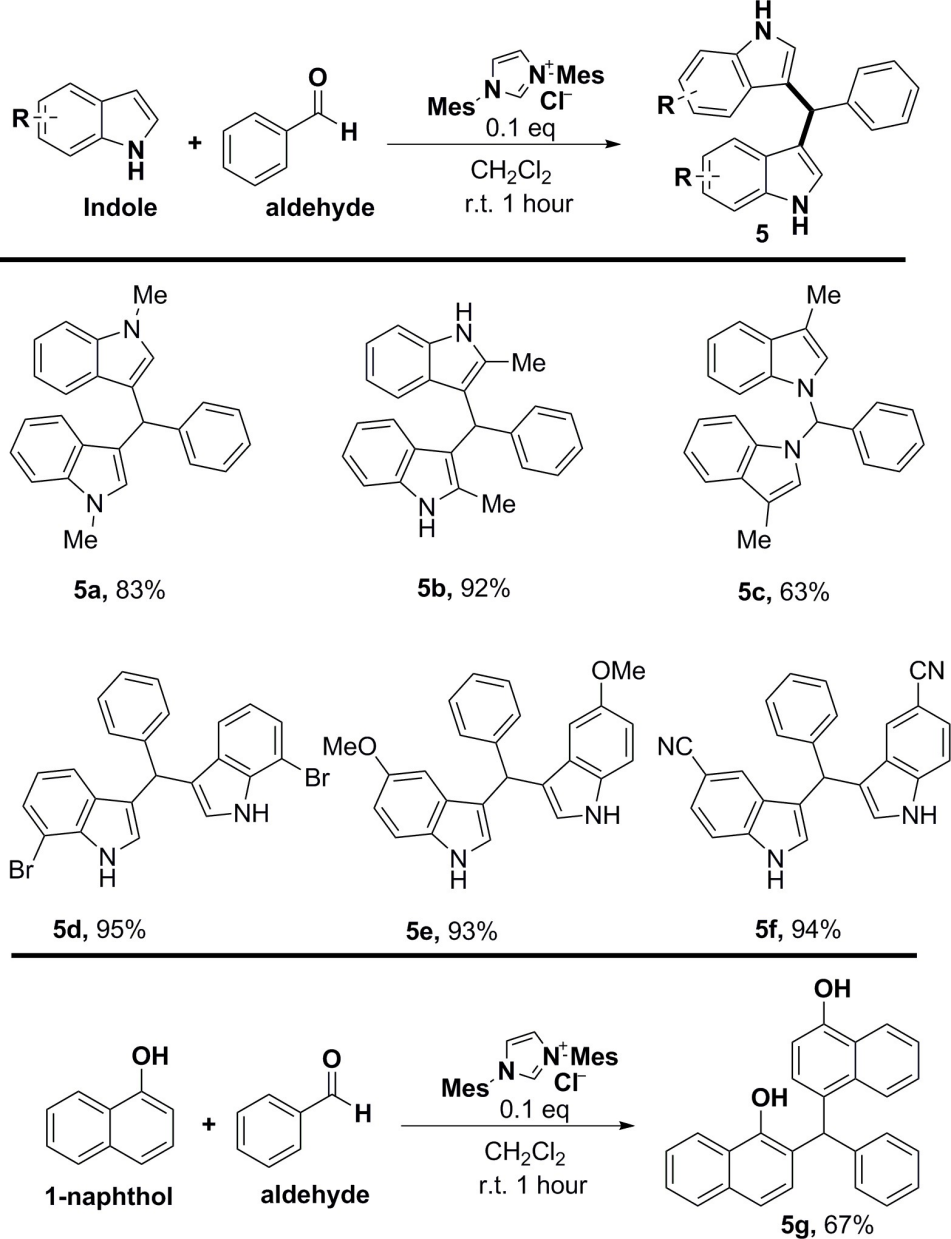
Scope of indoles and naphthols^a, b^.

^a^ Reaction conditions: A mixture of aldehyde (0.5 mmol, 1.0 equiv), catalyst (10 mol%, 0.2 equiv), indole (1.0 mmol, 2.0 equiv), in solvent (5.0 mL) was stirred at room temperature for 1h. ^b^ Isolated yield after flash chromatography.

The generality of the reaction with respect to the substituents on the indoles was also investigated (Fig 5). The methyl-substituted indoles at the 1- or 2-position did not affect the reaction due to the protection of the nitrogen or steric hindrance. On the contrary, the corresponding products were afforded in high yields (**5a** and **5b**). Surprisingly, the 3-methyl indole provided an addition product at the 1-position in a moderate yield (**5c**). The result differs from the known method of regioselectively producing (2,2′-bis-3-methylindolyl)methanes using ionic liquids under microwave irradiation condition, [38] possibly due to the catalytic process mediated by the cation-π interaction of an indole/imidazolium complex. The structure of **5c** was determined by two dimensional NMR (HSQC, HMBC. S1 File). Both electron-donating and electron-withdrawing substituents were accommodated on the indole ring furnishing excellent yields (**5d**–**5f**). 1-Naphthol was also explored as alternate nucleophilic substrate and yielded **5g** through a dual-addition reaction at the C2 and C4 of 2-naphthol, respectively. The product was confirmed by comparison to the reported NMR data for this compound, prepared through an alternate route.[39]

**Fig 6:**
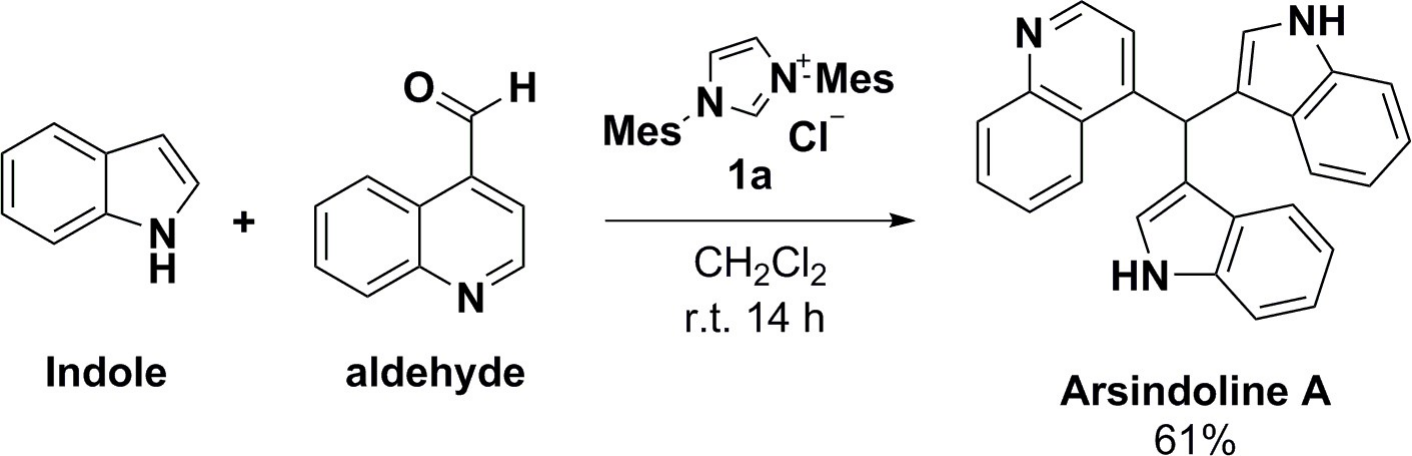
Synthesis of arsindoline A.

Meanwhile, we used this method to synthesize the natural product arsindoline A. Although the reaction time was prolonged and the yield was moderate, this effectively expands the scope of heterocyclic substrate. (Fig 6)

**Fig 7:**
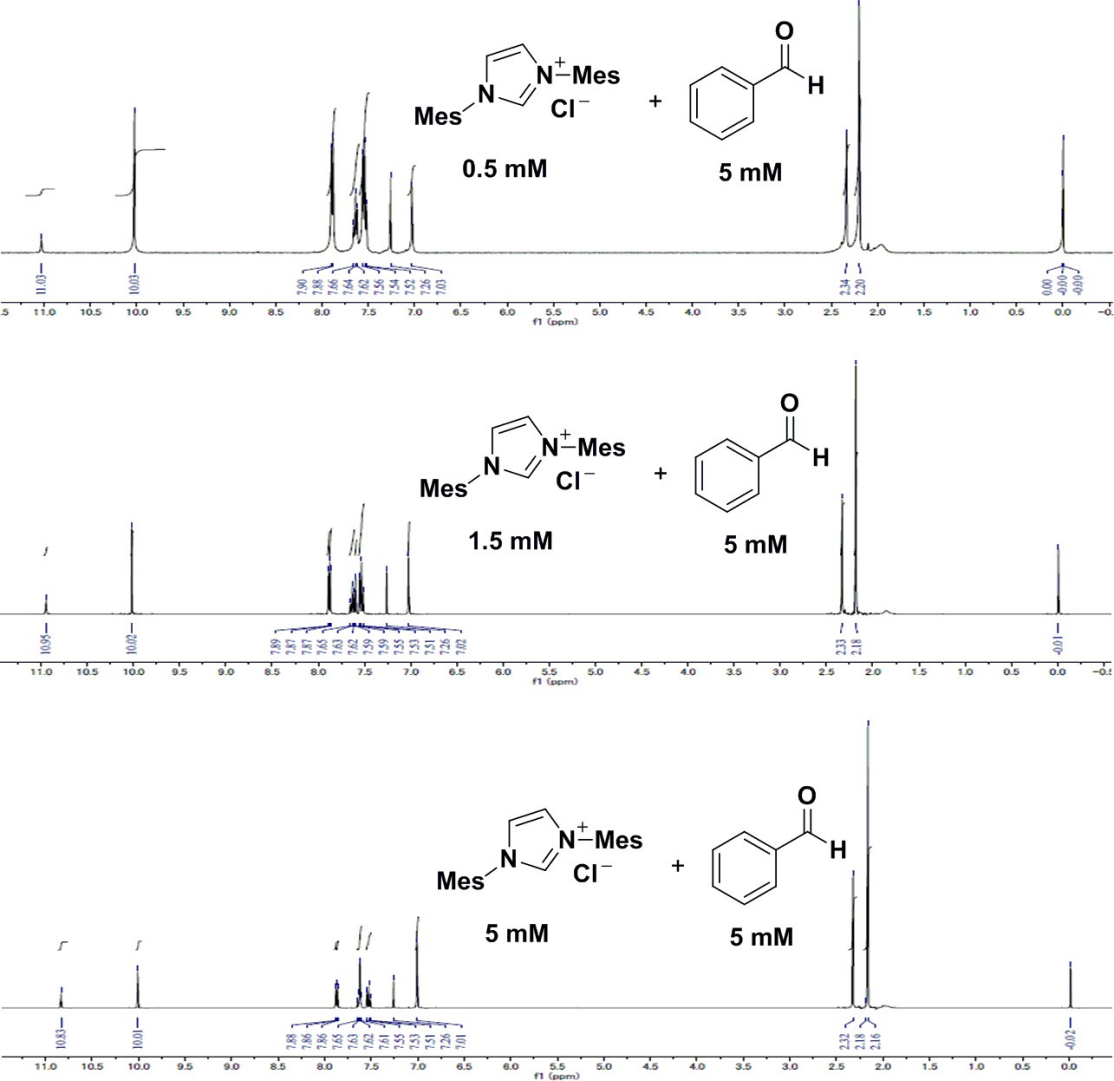
^1^H-NMR investigation of the interaction of IMesCl (1a) and benzaldehyde (3) in CDCl_3_.

In their previous report, Tamamura and co-workers showed that imidazolium salts activated indoles through a cation-π interaction by ^1^H NMR and deuterium labeling studies.[33] To confirm the imidazolium salts were not activating benzaldehyde, we monitored the chemical shift of the C2 proton of the imidazolium salt and the CHO proton of the aldehyde. However, a significant change was not observed by ^1^H NMR (Fig 7) indicating the aldehyde did not interact with the imidazolium salts.

**Fig 8:**
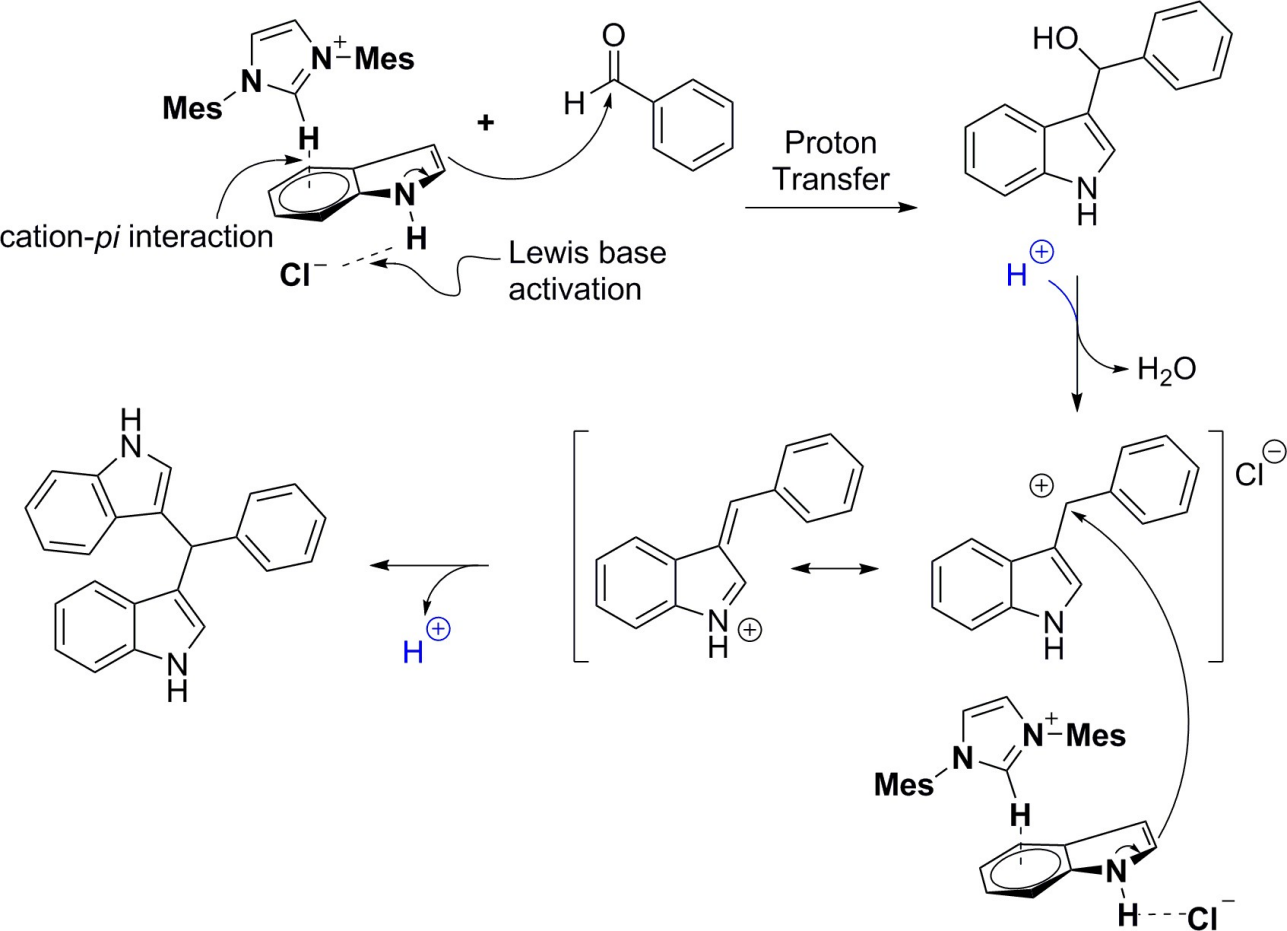
Proposed reaction mechanism.

A plausible reaction mechanism is shown in Fig 8. Based on the results of Tamamura’s mechanistic studies, we propose a catalytic process involving cation–π interaction of an indole/imidazolium complex, which increases the acidity of the indole, enabling deprotonating of the complex by the chloride anion. The intermediate (1*H*-indol-3-yl)(aryl)methanol is expected to ionize to an indolylphenylmethyl cationic species based on literature precedent,[40–42] which rapidly reacts with another molecule of indole to furnish the isolated bis(indolyl)methanes products. The inability to isolate the intermediate (1*H*-indol-3-yl)(aryl)methanol suggests the second step is very rapid.

## Conclusions

Although imidazolium salts are often used as precursors for NHC catalysts, the reaction of imidazolium salts as organocatalysts has rarely been applied. Herein, we have shown imidazolium salts provide a mild and efficient catalytic system for the electrophilic substitution reactions of indoles with a variety of carbonyl compounds to afford bis(indolyl)methanes. Furthermore, this method tolerates a wider substrate scope than other reactions to this important class of compounds and even allows utilization of 3-methyl-1*H*-indole and 1-naphthol nucleophiles. In summary, we have further expanded the reaction scope of imidazolium salt catalyzed dual activation addition reactions.

## Materials and methods

### 4.1. General information

Chemicals, catalysts and solvents were purchased from commercial suppliers and used as received. ^1^H, ^13^C spectra were recorded on a Bruker AVANCE III 400 (400 MHz), JEOL ECZ 400S (400 MHz) spectrometer. Chemical shifts were reported in parts per million (ppm), and the residual solvent peak was used as an internal reference: proton (CDCl_3_ δ 7.26), carbon (CDCl_3_ δ 77.16) was used as a reference. Multiplicity was indicated as follows: s (singlet), d (doublet), t (triplet), q (quartet), m (multiplet), dd (doublet of doublet), br s (broad singlet). Coupling constants were reported in Hertz (Hz). Coupling constants, J, are quoted in Hz and recorded to the nearest 0.1 Hz. Assignments (detailed in the supporting information) were confirmed using Distortionless Enhanced Polarisation Transfer NMR (DEPT 135) and two dimensional NMR Heteronuclear Single Quantum Coherence (HSQC) and Hetero-nuclear Multiple Bond Correlation (HMBC)) experiments gave information used to assign both the ^1^H NMR and ^13^C NMR spectra. High resolution mass spectrometry (HRMS) was performed using positive/negative electrospray ionisation (ESI+/ESI-), on Thermofisher Exactive Plus mass spectrometer. All m/z values are reported to 4 decimal places and are within ±5 ppm of theoretical values. Melting points were recorded on a Kofler hot block and are uncorrected. For thin layer chromatography (TLC), Merck pre-coated TLC plates (Merck 60 F254) were used, and compounds were visualized with a UV light at 254 nm. Flash chromatography separations were performed on Merck 60 (0.040–0.063 mm) mesh silica gel.

### 4.2. General procedure for the synthesis of bis(indolyl)methanes and bis(naphthyl)methane

An oven-dried 20 mL Schlenk flask was charged with triazolium salt (0.1 mmol, 0.2 equiv), indole derivatives or naphthol (1.0 mmol, 2.0 equiv). A solution of the aldehyde (0.5 mmol, 1.0 equiv, 5.0 mL, 0.1 M in CH_2_Cl_2_) was then added via syringe. The reaction mixture was stirred at room temperature for 1 h. Next, the reaction was quenched with water and the aqueous mixture was extracted with EtOAc (10 mL × 3). The extracts were combined, dried with MgSO_4_, and concentrated to afford the residue, which was chromatographed on silica gel (15 g, 20:1–3:1 PE/EA) to give bis(indolyl)methanes or bis(naphthyl)methane.

3,3'-(Phenylmethylene)bis(1*H*-indole) (**4a**). Condensation of indole and benzaldehyde using the general method afforded the title compound (154 mg, yield 95%) as a red solid. *R*_*f*_ = 0.30 (40:10 PE:EA); mp 139–140 ^o^C, lit. mp 141–142 ^o^C [43]; ^1^H NMR (400 MHz, CDCl_3_) δ 5.89 (1H, s), 6.67 (2H, dd, *J* = 2.4, 0.9 Hz), 7.00 (2H, td, *J* = 7.5, 1.0 Hz), 7.19 (2H, t, *J* = 7.9 Hz), 7.22–7.25 (1H, m), 7.28–7.30 (2H, m), 7.34–7.39 (4H, m), 7.40–7.43 (2H, m), 7.90 (2H, br s); ^13^C NMR (100 MHz, CDCl_3_) δ 40.2, 111.0, 119.3, 119.8, 120.0, 121.9, 123.6, 126.2, 127.1, 128.2, 128.7, 136.7, 144.0; HRMS (ESI+) calcd for C_23_H_19_N_2_ [M+H]^+^ 323.1543, found 323.1531 (error 3.7 ppm). All spectroscopic data were in agreement with the literature values. [20]

3,3'-((4-Ethylphenyl)methylene)bis(1*H*-indole) (**4b**). Condensation of indole and benzaldehyde using the general method afforded the title compound (168 mg, yield 96%) as a white solid. *R*_*f*_ = 0.33 (40:10 PE:EA); mp 161–163 ^o^C; ^1^H NMR (400 MHz, CDCl_3_) δ 1.22 (3H, t, *J* = 8.5 Hz), 2.61 (2H, q, *J* = 8.5 Hz), 5.86 (1H, s), 6.66 (2H, s), 7.00 (2H, t, *J* = 7.5 Hz), 7.11 (2H, d, *J* = 9.0 Hz), 7.18 (2H, t, *J* = 7.5 Hz), 7.26 (2H, d, *J* = 9.0 Hz), 7.35 (2H, d, *J* = 7.5 Hz), 7.40 (2H, d, *J* = 7.5 Hz), 7.86 (2H, br s); ^13^C NMR (100 MHz, CDCl_3_) δ 15.5, 28.5, 39.8, 111.0, 119.2, 119.99, 120.01, 121.9, 123.6, 127.1, 127.7, 128.6, 136.7, 141.2, 141.9; HRMS (ESI+) calcd for C_25_H_23_N_2_ [M+H]^+^ 351.1840, found 351.1855 (error 4.3 ppm).

3,3'-([1,1'-Biphenyl]-4-ylmethylene)bis(1*H*-indole) (**4c**). Condensation of indole and benzaldehyde using the general method afforded the title compound (183 mg, yield 92%) as a pink solid. *R*_*f*_ = 0.34 (40:10 PE:EA); mp 230–232 ^o^C, lit. mp 241–243 ^o^C [44]; ^1^H NMR (400 MHz, CDCl_3_) δ 5.94 (1H, s), 6.62–6.67 (2H, m), 7.02 (2H, t, *J* = 8.0 Hz), 7.18 (2H, t, *J* = 7.6 Hz), 7.32 (1H, t, *J* = 7.3 Hz), 7.34–7.39 (3H, m), 7.40–7.48 (5H, m), 7.52 (2H, d, *J* = 8.9 Hz), 7.59 (2H, d, *J* = 8.9 Hz), 7.92 (2H, br s); ^13^C NMR (100 MHz, CDCl_3_) δ 39.9, 111.1, 119.3, 119.7, 120.0, 122.0, 123.6, 126.95, 126.99 (2C), 127.1, 128.7, 129.1, 136.7, 138.9, 141.1, 143.2; HRMS (ESI-) calcd for C_29_H_21_N_2_ [M−H]^−^ 397.1684, found 397.1699 (error 3.8 ppm). All spectroscopic data were in agreement with the literature values. [44]

3,3'-((2-Fluorophenyl)methylene)bis(1*H*-indole) (**4d**). Condensation of indole and benzaldehyde using the general method afforded the title compound (157 mg, yield 92%) as a red solid. *R*_*f*_ = 0.35 (40:10 PE:EA); mp 80–81 ^o^C, lit. mp 77 ^o^C [28]; ^1^H NMR (400 MHz, CDCl_3_) δ 6.24 (1H, s), 6.70 (2H, s), 6.98–7.04 (3H, m), 7.08 (1H, t, *J* = 9.2 Hz), 7.16–7.22 (4H, m), 7.36 (2H, d, *J* = 8.2 Hz), 7.41 (2H, d, *J* = 7.9 Hz), 7.89 (2H, br s); ^13^C NMR (100 MHz, CDCl_3_) δ 32.5(d, *J* = 4.1 Hz), 111.1, 115.3 (d, *J* = 22.4 Hz), 118.3, 119.3, 119.8, 122.0, 123.6, 123.9 (d, *J* = 3.5 Hz), 126.9, 127.8 (d, *J* = 8.1 Hz), 130.4 (d, *J* = 3.5 Hz), 130.9 (d, *J* = 14.0 Hz), 136.7, 160.7 (d, *J* = 245.9 Hz); HRMS (ESI+) calcd for C_23_H_18_FN_2_ [M+H]^+^ 341.1449, found 341.1437 (error 3.5 ppm). All spectroscopic data were in agreement with the literature values. [28]

3,3'-((2-Bromo-6-fluorophenyl)methylene)bis(1*H*-indole) (**4e**). Condensation of indole and benzaldehyde using the general method afforded the title compound (194 mg, yield 93%) as a red solid. *R*_*f*_ = 0.32 (40:10 PE:EA); mp 113–115 ^o^C; ^1^H NMR (400 MHz, CDCl_3_) δ 6.27 (1H, s), 6.64 (2H, dd, *J* = 2.3, 1.0 Hz), 6.83 (1H, ddd, *J* = 8.7, 7.8, 3.1 Hz), 6.95 (1H, dd, *J* = 9.8, 3.1 Hz), 7.04 (2H, t, *J* = 7.2 Hz), 7.21 (2H, t, *J* = 7.2 Hz), 7.41–7.39 (4H, m), 7.51–7.61 (1H, m), 7.95 (2H, br s); ^13^C NMR (100 MHz, CDCl_3_) δ 39.8, 111.2, 115.2 (d, *J* = 22.4 Hz), 117.8 (d, *J* = 23.5 Hz), 117.9, 118.8 (d, *J* = 3.0 Hz), 119.5, 119.8, 122.2, 123.8, 126.9, 133.9 (d, *J* = 7.8 Hz), 136.8, 145.5 (d, *J* = 6.6 Hz), 162.1 (d, *J* = 245.8 Hz); HRMS (ESI+) calcd for C_23_H_17_FBrN_2_ [M+H]^+^ 419.2889, found 419.2894 (error 1.2 ppm).

3,3'-((3-Hydroxyphenyl)methylene)bis(1*H*-indole) (**4f**). Condensation of indole and benzaldehyde using the general method afforded the title compound (152 mg, yield 90%) as a red solid. *R*_*f*_ = 0.20 (40:10 PE:EA); mp 105–107 ^o^C, lit. mp 100 ^o^C [21]; ^1^H NMR (400 MHz, CDCl_3_) δ 5.35 (1H, s), 6.00 (1H, s), 6.78 (2H, d, *J* = 2.4 Hz), 6.86 (2H, t, *J* = 7.5 Hz), 7.02 (2H, t, *J* = 7.5 Hz), 7.13–7.23 (4H, m), 7.28 (4H, dd, *J* = 10.8, 8.4 Hz), 7.99 (2H, br s); ^13^C NMR (100 MHz, CDCl_3_) δ 35.9, 111.2, 116.7, 117.2, 119.6, 119.9, 120.8, 122.4, 123.6, 126.9, 128.1, 129.1, 130.0, 136.9, 154.6; HRMS (ESI+) calcd for C_23_H_19_N_2_O [M+H]^+^ 339.1483, found 339.1492 (error 2.7 ppm). All spectroscopic data were in agreement with the literature values. [21]

3,3'-((4-Methoxyphenyl)methylene)bis(1*H*-indole) (**4g**). Condensation of indole and benzaldehyde using the general method afforded the title compound (149 mg, yield 85%) as a pink solid. *R*_*f*_ = 0.35 (40:10 PE:EA); mp 213–215 ^o^C, lit. mp 217–219 ^o^C [28]; ^1^H NMR (400 MHz, CDCl_3_) δ 3.79 (3H, s), 5.84 (1H, s), 6.65 (2H, dd, *J* = 2.4, 1.0 Hz), 6.81–6.84 (2H, m), 6.97–7.02 (2H, m), 7.14–7.20 (2H, m), 7.24–7.27 (2H, m), 7.34–7.36 (2H, m), 7.38–7.41 (2H, m), 7.88 (2H, br s); ^13^C NMR (100 MHz, CDCl_3_) δ 39.4, 55.2, 111.0, 113.6, 119.2, 120.0, 120.1, 121.9, 123.5, 127.1, 129.6, 136.2, 136.7, 157.9; HRMS (ESI-) calcd for C_24_H_19_N_2_O [M−H]^−^ 351.1491, found 351.1482 (error 2.6 ppm). All spectroscopic data were in agreement with the literature values.[28]

3,3'-((3-Phenoxyphenyl)methylene)bis(1*H*-indole) (**4h**). Condensation of indole and benzaldehyde using the general method afforded the title compound (182 mg, yield 88%) as a red solid. *R*_*f*_ = 0.34 (40:10 PE:EA); mp 82–83 ^o^C, lit. mp 84–86 ^o^C [13]; ^1^H NMR (400 MHz, CDCl_3_) δ 5.87 (1H, s), 6.68 (2H, d, *J* = 2.4 Hz), 6.86 (1H, dd, *J* = 8.0, 2.4 Hz), 6.92 (2H, d, *J* = 8.0 Hz), 6.98–7.08 (4H, m), 7.09–7.22 (3H, m), 7.23–7.27 (3H, m), 7.35 (2H, d, *J* = 8.1 Hz), 7.40 (2H, d, *J* = 7.9 Hz), 7.89 (2H, br s); ^13^C NMR (100 MHz, CDCl_3_) δ 40.2, 111.1, 116.9, 118.3, 119.3, 119.4, 119.8, 119.9, 122.0, 122.8, 123.5, 124.0, 127.0, 129.5, 129.6, 136.7, 146.3, 156.9, 157.5; HRMS (ESI+) calcd for C_29_H_23_N_2_O [M+H]^+^ 415.1787, found 415.1805 (error 4.3 ppm). All spectroscopic data were in agreement with the literature values. [13]

3,3'-((4-Nitrophenyl)methylene)bis(1*H*-indole) (**4i**). Condensation of indole and benzaldehyde using the general method afforded the title compound (174 mg, yield 95%) as a red solid. *R*_*f*_ = 0.30 (40:10 PE:EA); mp 208–210 ^o^C, lit. mp 222–224 ^o^C [29]; ^1^H NMR (400 MHz, CDCl_3_) δ 6.00 (1H, s), 6.69 (2H, d, *J* = 2.4 Hz), 7.03 (2H, t, *J* = 7.5 Hz), 7.20 (2H, t, *J* = 7.6 Hz, 2H), 7.34 (2H, d, *J* = 8.0 Hz), 7.39 (2H, d, *J* = 8.1 Hz), 7.51 (2H, d, *J* = 8.3 Hz), 8.00 (2H, s), 8.14 (2H, d, *J* = 8.3 Hz); ^13^C NMR (100 MHz, CDCl_3_) δ 40.2, 111.3, 118.2, 119.58, 119.64, 122.4, 123.7 (2C), 126.7, 129.5, 136.7, 146.6, 151.8; HRMS (ESI-) calcd for C_23_H_16_N_3_O_2_ [M−H]^−^ 366.1226, found 366.1237 (error 3.0 ppm). All spectroscopic data were in agreement with the literature values. [29]

3,3'-((4-Cyanophenyl)methylene)bis(1*H*-indole) (**4j**). Condensation of indole and benzaldehyde using the general method afforded the title compound (163 mg, yield 94%) as an orange solid. *R*_*f*_ = 0.32 (40:10 PE:EA); mp 209–210 ^o^C, lit. mp 213–215 ^o^C[29]; ^1^H NMR (400 MHz, CDCl_3_) δ 6.94 (1H, s), 6.66 (2H, d, *J* = 2.4 Hz), 7.03 (2H, t, *J* = 7.5 Hz), 7.20 (2H, t, *J* = 7.6 Hz, 2H), 7.33 (2H, d, *J* = 8.0 Hz), 7.38 (2H, d, *J* = 8.2 Hz), 7.45 (2H, d, *J* = 8.0 Hz), 7.57 (2H, d, *J* = 8.0 Hz), 7.98 (2H, s); ^13^C NMR (100 MHz, CDCl_3_) δ 40.4, 110.1, 111.3, 118.3, 119.2, 119.60, 119.63, 122.3, 123.7, 126.7, 129.5, 132.2, 136.7, 149.8; HRMS (ESI-) calcd for C_24_H_16_N_3_ [M−H]^−^ 346.1324, found 346.1339 (error 4.3 ppm). All spectroscopic data were in agreement with the literature values. [16]

3,3'-(Heptane-1,1-diyl)bis(1*H*-indole) (**4k**). Condensation of indole and benzaldehyde using the general method afforded the title compound (140 mg, yield 85%) as a white solid. *R*_*f*_ = 0.33 (40:10 PE:EA); mp 74–75 ^o^C, lit. mp 71–72 ^o^C [45]; ^1^H NMR (400 MHz, CDCl_3_) δ 0.80–0.90 (3H, m), 1.24–1.44 (8H, m), 2.20–2.38 (2H, m), 4.48 (1H, t, *J* = 6.0 Hz), 6.99 (2H, d, *J* = 2.2 Hz), 7.04 (2H, t, *J* = 7.5 Hz), 7.15 (2H, t, *J* = 7.6 Hz), 7.33 (2H, d, *J* = 8.0 Hz), 7.61 (2H, d, *J* = 7.9 Hz), 7.86 (2H, br s); ^13^C NMR (100 MHz, CDCl_3_) δ 22.7, 28.3, 29.5, 31.8, 31.9, 34.0, 111.0, 119.0, 119.7, 120.7, 121.4, 121.7, 127.7, 136.6; HRMS (ESI-) calcd for C_23_H_25_N_2_ [M−H]^−^ 329.1999, found 329.2012 (error 3.9 ppm). All spectroscopic data were in agreement with the literature values. [45]

3,3'-(Cyclohexylmethylene)bis(1*H*-indole) (**4l**). Condensation of indole and benzaldehyde using the general method afforded the title compound (136 mg, yield 83%) as a white solid. *R*_*f*_ = 0.35 (40:10 PE:EA); mp 115–117 ^o^C, lit. mp 117–119 ^o^C [23]; ^1^H NMR (400 MHz, CDCl_3_) δ 1.10–1.20 (3H, m), 1.22–1.26 (2H, m), 1.61–1.70 (3H, m), 1.79–1.86 (2H, m), 2.22–2.29 (1H, m), 4.48 (1H, d, *J* = 8.8 Hz), 7.05 (2H, t, *J* = 7.6 Hz), 7.10 (2H, d, *J* = 2.3 Hz), 7.13 (2H, t, *J* = 7.6 Hz), 7.31 (2H, d, *J* = 8.1 Hz), 7.66 (2H, d, *J* = 7.9 Hz), 7.88 (2H, br s); ^13^C NMR (100 MHz, CDCl_3_) δ 26.7, 26.7, 32.4, 40.2, 43.0, 111.0, 119.0, 119.69, 119.72, 121.5, 121.6, 127.8, 136.3; HRMS (ESI-) calcd for C_23_H_23_N_2_ [M−H]^−^ 327.1842, found 327.1856 (error 4.3 ppm). All spectroscopic data were in agreement with the literature values. [23]

3,3'-(Acetylmethylene)bis(1*H*-indole) (**4m**). Condensation of indole and benzaldehyde using the general method afforded the title compound (93 mg, yield 65%) as a yellow solid. *R*_*f*_ = 0.31 (40:10 PE:EA); mp 111–113 ^o^C; ^1^H NMR (400 MHz, CDCl_3_) δ 2.33 (3H, s), 5.57 (1H, s), 7.08–7.12 (4H, m), 7.20 (2H, t, *J* = 7.6Hz), 7.37 (2H, d, *J* = 8.1Hz), 7.55 (2H, d, *J* = 8.0Hz), 8.07 (2H, br); ^13^C NMR (100 MHz, CDCl_3_) δ 28.9, 48.1, 111.2, 113.6, 119.2, 119.7, 122.3, 123.3, 126.8, 136.3, 207.0; HRMS (ESI+) calcd for C_19_H_17_N_2_O [M+H]^+^ 289.1324, found 289.1335 (error 3.8 ppm). All spectroscopic data were in agreement with the literature values. [46]

(*E*)-3,3'-(3-(4-Fluorophenyl)prop-2-ene-1,1-diyl)bis(1*H*-indole) (**4n**). Condensation of indole and benzaldehyde using the general method afforded the title compound (159 mg, yield 87%) as a yellow solid. *R*_*f*_ = 0.33 (40:10 PE:EA); mp 125–127 ^o^C; ^1^H NMR (400 MHz, CDCl_3_) δ 5.40 (1H, dd, *J* = 7.0, 1.2 Hz), 6.50 (1H, dd, *J* = 15.8, 1.2 Hz), 6.72 (1H, ddd, *J* = 15.8, 7.0, 0.5 Hz), 6.87–7.00 (4H, m), 7.09 (2H, t, *J* = 8.0 Hz), 7.19 (2H, t, *J* = 8.2 Hz), 7.27–7.36 (2H, m), 7.38 (2H, dt, *J* = 8.0, 0.9 Hz), 7.59 (2H, dq, *J* = 8.0, 0.9 Hz), 7.96 (2H, br s); ^13^C NMR (100 MHz, CDCl_3_) δ 37.5, 111.1, 115.3 (d, *J* = 21.4 Hz), 118.4, 119.3, 112.0, 122.0, 122.5, 127.0, 127.8 (d, *J* = 8.0 Hz), 128.8, 132.1 (d, *J* = 2.1 Hz), 133.9 (d, *J* = 3.0 Hz), 136.7, 162.0 (d, *J* = 245.9 Hz); HRMS (ESI-) calcd for C_25_H_18_N_2_F [M−H]^−^ 365.1439, found 365.1449 (error 2.7 ppm).

(*E*/*Z*)-3,3'-(3,7-Dimethylocta-2,6-diene-1,1-diyl)bis(1*H*-indole) (**4o**). Condensation of indole and benzaldehyde using the general method afforded the title compound (149 mg, yield 81%, *E*/*Z* 3:2) as a yellow solid. *R*_*f*_ = 0.35 (40:10 PE:EA); mp 101–103 ^o^C; ^1^H NMR (400 MHz, CDCl_3_) δ 1.56 and 1.60 (3H, s), 1.62 and 1.68 (3H, s), 1.80 and 1.85 (3H, s), 2.12–2.33 (4H, m), 5.12–5.15 (1H, m), 5.37–5.42 (1H, m), 5.72–5.77 (1H, m), 6.86–6.95 (2H, m), 7.01–7.08 (2H, m), 7.12–7.19(2H, m), 7.31–7.38 (2H, m), 7.52–7.58 (2H, m), 7.86 (2H, br s); ^13^C NMR (100 MHz, CDCl_3_) δ 16.4 and 17.8, 17.9 and 18.4, 23.5 and 25.9, 25.8 and 26.7, 32.4 and 33.3, 33.1 and 39.8, 111.2, 119.06 and 119.08, 119.8 and 120.0, 120.0 and 120.2, 121.78 and 121.81, 122.15 and 122.17, 124.5 and 124.6, 127.1 and 127.2, 128.1 and 128.6, 131.5 and 131.8, 134.6 and 134.8, 136.80 and 136.82; HRMS (ESI+) calcd for C_26_H_29_N_2_ [M+H]^+^ 369.2311, found 369.2325 (error 3.8 ppm).

3,3',3''-(Propane-1,1,3-triyl)tris(1*H*-indole) (**4p**). Condensation of indole and benzaldehyde using the general method afforded the title compound (177 mg, yield 83%) as a yellow solid. *R*_*f*_ = 0.31 (40:10 PE:EA); mp 179–181 ^o^C, lit. mp 180–185 ^o^C [47]; ^1^H NMR (400 MHz, CDCl_3_) δ 2.63–2.70 (2H, m), 2.86–2.91 (2H, m), 4.58–4.63 (1H, m), 6.93–6.94 (1H, m), 7.00–7.11 (5H, m), 7.14–7.20 (3H, m), 7.32–7.36 (3H, m), 7.50–7.58 (3H, m), 7.86 (1H, br s), 7.90 (2H, br s); ^13^C NMR (100 MHz, CDCl_3_) δ 23.8, 33.7, 35.9, 110.95, 111.00, 116.8, 119.00, 119.02, 119.1, 119.7, 120.2, 121.1, 121.5, 121.7, 121.8, 127.1, 127.6, 136.3, 136.6; HRMS (ESI+) calcd for C_27_H_24_N_3_ [M+H]^+^ 390.1951, found 390.1964 (error 3.3 ppm). All spectroscopic data were in agreement with the literature values.[47]

3,3'-(Phenylmethylene)bis(1-methyl-1*H*-indole) (**5a**). Condensation of indole and benzaldehyde using the general method afforded the title compound (146 mg, yield 83%) as a pink solid. *R*_*f*_ = 0.61 (40:10 PE:EA); mp 181–183 ^o^C, lit. mp 180–183 ^o^C [48]; ^1^H NMR (400 MHz, CDCl_3_) δ 3.69 (6H, s), 5.90 (1H, s), 6.55 (2H, s), 6.99–7.03 (2H, m), 7.18–7.25 (3H, m), 7.27–7.32 (4H, m), 7.32–7.42 (4H, m); ^13^C NMR (100 MHz, CDCl_3_) δ 32.7, 40.1, 109.1, 118.3, 118.7, 120.1, 121.4, 126.0, 127.5, 128.2, 128.3, 128.7, 137.4, 144.5; HRMS (ESI-) calcd for C_25_H_21_N_2_ [M−H]^−^ 349.1690, found 349.1699 (error 2.6 ppm). All spectroscopic data were in agreement with the literature values. [48]

3,3'-(Phenylmethylene)bis(2-methyl-1*H*-indole) (**5b**). Condensation of indole and benzaldehyde using the general method afforded the title compound (161 mg, yield 92%) as a pink solid. *R*_*f*_ = 0.39 (40:10 PE:EA); mp 249–250 ^o^C, lit. mp 257–258 ^o^C [49]; ^1^H NMR (400 MHz, CDCl_3_) δ 2.06 (6H, s), 6.01 (1H, s), 6.82–6.88 (2H, m), 6.98 (2H, d, *J* = 8.7Hz), 7.02–7.08 (2H, m), 7.20–7.30 (8H, m), 7.72 (2H, br s); ^13^C NMR (100 MHz, CDCl_3_) δ 12.5, 39.2, 110.0, 113.4, 119.1, 119.4, 120.6, 126.0, 128.1, 129.0, 129.1, 131.8, 135.0, 143.7; HRMS (ESI-) calcd for C_25_H_21_N_2_ [M−H]^−^ 349.1690, found 349.1699 (error 2.6 ppm). All spectroscopic data were in agreement with the literature values. [49]

1,1'-(Phenylmethylene)bis(3-methyl-1*H*-indole) (**5c**). Condensation of indole and benzaldehyde using the general method afforded the title compound (110 mg, yield 63%) as a white solid. *R*_*f*_ = 0.55 (40:10 PE:EA); mp 110–112 ^o^C; ^1^H NMR (400 MHz, CDCl_3_) δ 2.26 (6H, s), 6.58 (2H, s), 7.03–7.10 (2H, m), 7.12–7.18 (4H, m), 7.20–7.24 (2H, m), 7.26–7.27 (1H, m), 7.30–7.37 (3H, m), 7.56–7.62 (2H, m), 7.87 (1H, s); ^13^C NMR (100 MHz, CDCl_3_) δ 9.8, 68.5, 109.5, 112.0, 119.3, 119.8, 122.3, 123.0, 127.3, 129.0, 129.1, 129.5, 136.4, 137.1; HRMS (ESI+) calcd for C_25_H_23_N_2_ [M+H]^+^ 351.1843, found 351.1855 (error 3.4 ppm).

3,3'-(Phenylmethylene)bis(7-bromo-1*H*-indole) (**5d**). Condensation of indole and benzaldehyde using the general method afforded the title compound (228 mg, yield 95%) as a pink solid. *R*_*f*_ = 0.32 (40:10 PE:EA); mp 239–241 ^o^C; ^1^H NMR (400 MHz, CDCl_3_) δ 5.80 (1H, s), 6.63 (2H, dd, *J* = 2.3, 1.3 Hz), 7.03–7.11 (2H, m), 7.20 (2H, d, *J* = 8.3Hz), 7.27–7.30 (5H, m), 7.51 (2H, d, *J* = 2.3Hz), 7.93 (2H, br); ^13^C NMR (100 MHz, CDCl_3_) δ 40.0, 114.0, 115.7, 119.7, 121.1, 122.7, 124.1, 125.9, 126.5, 128.4, 128.6, 137.5, 143.3; HRMS (ESI+) calcd for C_25_H_15_N_2_Br_2_ [M+H]^+^ 478.9568, found 478.9576 (error 1.7 ppm).

3,3'-(Phenylmethylene)bis(5-methoxy-1*H*-indole) (**5e**). Condensation of indole and benzaldehyde using the general method afforded the title compound (177 mg, yield 93%) as a white solid. *R*_*f*_ = 0.35 (40:10 PE:EA); mp 213–215 ^o^C, lit. mp 218–220 ^o^C [20]; ^1^H NMR (400 MHz, CDCl_3_) δ 3.69 (6H, s), 5.77 (1H, s), 6.66–6.67 (2H, m), 6.79–6.80 (2H, m), 6.82 (1H, d, *J* = 2.5 Hz), 6.84 (1H, d, *J* = 2.5 Hz), 7.20–7.22 (1H, m), 7.23–7.25 (2H, m), 7.27–7.30 (2H, m), 7.32–7.36 (2H, m), 7.81 (2H, br s); ^13^C NMR (100 MHz, CDCl_3_) δ 40.3, 55.9, 102.0, 111.7, 111.9, 119.3, 124.4, 126.1, 127.5, 128.2, 128.7, 131.9, 143.9, 153.7; HRMS (ESI+) calcd for C_25_H_23_N_2_O_2_ [M+H]^+^ 383.1740, found 383.1754 (error 3.7 ppm). All spectroscopic data were in agreement with the literature values. [20]

3,3'-(Phenylmethylene)bis(1*H*-indole-5-carbonitrile) (**5f**). Condensation of indole and benzaldehyde using the general method afforded the title compound (171 mg, yield 94%) as a pink solid. *R*_*f*_ = 0.32 (40:10 PE:EA); mp 243–244 ^o^C, lit. mp 241–243 ^o^C [27]; ^1^H NMR (400 MHz, CDCl_3_) δ 5.83 (1H, s), 6.81 (2H, dd, *J* = 2.5, 1.2 Hz), 7.26–7.30 (5H, m), 7.40–7.46 (4H, m), 7.66 (2H, d, *J* = 2.5 Hz), 8.41 (2H, br s); ^13^C NMR (100 MHz, CDCl_3_) δ 40.0, 102.6, 112.2, 119.9, 120.7, 125.2, 125.5, 125.6, 126.6, 127.0, 128.4, 128.7, 138.4, 142.3; HRMS (ESI+) calcd for C_25_H_17_N_4_ [M+H]^+^ 373.1442, found 373.1448 (error 1.6 ppm).

2-((4-Hydroxynaphthalenyl)(phenyl)methyl)naphthalen-1-ol (**5g**). Condensation of 1-naphthanol and benzaldehyde using the general method afforded the title compound (126 mg, yield 67%) as a white solid. *R*_*f*_ = 0.37 (10:10 PE:EA); mp 201–203 ^o^C, lit. mp 205–206 ^o^C [39]; ^1^H NMR (400 MHz, CDCl_3_) δ 5.34 (1H, br s), 5.64 (1H, br s), 6.42 (1H, s), 6.70 (1H, d, *J* = 7.8 Hz), 6.87 (1H, d, *J* = 7.8 Hz), 6.97 (1H, d, *J* = 9.5 Hz), 7.19–7.23 (2H, m), 7.25–7.38 (4H, m), 7.39–7.42 (1H, m), 7.44–7.50 (3H, m), 7.75–7.80 (1H, m), 7.90 (1H, d, *J* = 9.5 Hz), 8.13–8.17 (1H, m), 8.28 (1H, d, *J* = 11.4 Hz); ^13^C NMR (100 MHz, CDCl_3_) δ 48.0, 107.9, 120.4, 121.6, 122.5, 123.6, 124.2, 125.1, 125.1, 125.2, 125.4, 126.0, 127.1 (2C), 127.4, 127.6, 128.1, 128.9, 129.7, 130.1, 133.0, 133.7, 142.3, 148.8, 151.3; HRMS (ESI+) calcd for C_27_H_21_O_2_ [M+H]^+^ 377.1527, found 377.1536 (error 2.4 ppm). All spectroscopic data were in agreement with the literature values. [39]

4-(di(1*H*-indol-3-yl)methyl)quinoline (**arsindoline A**). Condensation of quinoline-4-carbaldehyde and indole using the general method afforded the title compound (114 mg, yield 61%) as a yellow solid. *R*_*f*_ = 0.54 (10:20 PE:EA); mp 169–170 ^o^C, lit. mp 164–168 ^o^C [50]; ^1^H NMR (500 MHz, CDCl_3_) δ 6.55 (s, 2H), 6.66 (s, 1H), 7.03 (t, *J* = 7.7 Hz, 2H), 7.15 (d, *J* = 4.5 Hz, 1H), 7.20 (t, *J* = 7.7 Hz, 2H), 7.35–7.40 (m, 4H), 7.43 (t, *J* = 7.7 Hz, 1H), 7.67 (t, *J* = 7.7 Hz, 1H), 8.13–8.17 (m, 4H), 8.73 (d, *J* = 4.5 Hz, 1H); ^13^C NMR (100 MHz, CDCl_3_) δ 35.6, 111.4, 117.6, 119.5 (2C), 121.1, 122.3, 124.3, 124.5, 126.7, 126.8, 127.5, 129.1, 129.9, 136.8, 148.4, 150.0, 150.4.; HRMS (ESI+) calcd for C_26_H_20_N_3_ [M+H]^+^ 374.1652, found 374.1658 (error 1.6 ppm). All spectroscopic data were in agreement with the literature values. [50]

## Supporting information

S1 File The file includes both the ^1^H NMR and ^13^C NMR spectra of all compounds and the two dimensional NMR spectra of compound 5c. (DOCX)
