# Supplementary material for: Development of an imidazole salt catalytic system for the preparation of bis(indolyl)methanes and bis(naphthyl)methane
Source: PLoS One. 2019 Apr 25;14(4):e0216008. doi: 10.1371/journal.pone.0216008 (PMC6483367; doi:10.1371/journal.pone.0216008)
Supplement: S1 File — (DOCX) [file pone.0216008.s001.docx]

**Supporting Information**

Xu Wang^a^ *, Courtney C. Aldrich^a, b^

^a^ Department of Synthetic Medicinal Chemistry, Institute of Materia Medica, Chinese Academy of Medical Sciences and Peking Union Medical College, Beijing 100050, People’s Republic of China

^b^ Department of Medicinal Chemistry, University of Minnesota, Minneapolis, MN 55455, USA

| **S1. ^1^H NMR, ^13^C NMR, DEPT and HSQC, HMBC...............…………** | **2** |
| --- | --- |

**S1. ^1^H NMR, ^13^C NMR, DEPT and HSQC, HMBC**

Compound **4a**

Compound **4b**

Compound **4c**

Compound **4d**

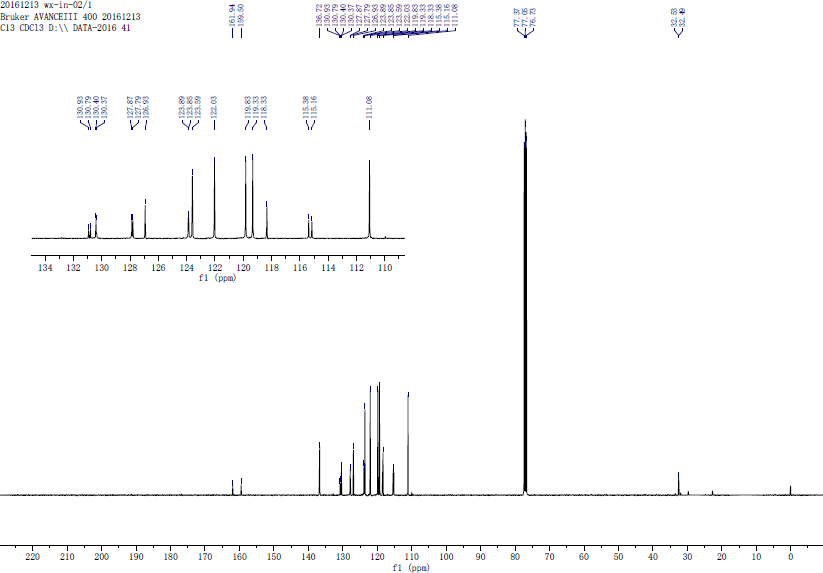


Compound **4e**

Compound **4f**

Compound **4g**

Compound **4h**

Compound **4i**

Compound **4j**

Compound **4k**

Compound **4l**

Compound **4m**

Compound **4n**

Compound **4o**

Compound **4p**

Compound **5a**

Compound **5b**

Compound **5c**

DEPT90

DEPT135

**HSQC**

**HMBC**

Compound **5d**

Compound **5e**

Compound **5f**

Compound **5g**

DEPT90

DEPT135
